# Proteotyping of *Campylobacter jejuni* by MALDI-TOF MS and Strain Solution Version 2 Software

**DOI:** 10.3390/microorganisms11010202

**Published:** 2023-01-12

**Authors:** Teruyo Ojima-Kato, Satomi Nagai, Akane Fujita, Junko Sakata, Hiroto Tamura

**Affiliations:** 1 School of Agriculture, Meijo University, Shiogamaguchi, Tenpaku-ku, Nagoya 468-0073, Japan; 2 Graduate School of Bioagricultural Sciences, Nagoya University, Furo-cho, Chikusa-ku, Nagoya 464-8601, Japan; 3 Osaka Institute of Public Health, Nakamichi, Higashinari-ku, Osaka 537-0025, Japan

**Keywords:** *Campylobacter jejuni*, MALDI-TOF MS, proteotyping, strain solution, theoretical mass database

## Abstract

Identification of microorganisms by MALDI-TOF MS has become a popular method in the past 20 years. Strain Solution ver. 2 software appended with MALDI-TOF MS enables accurate discrimination of serotypes and strains beyond the genus and species level by creating a theoretical mass-based database. In this study, we constructed a theoretical mass database with the validated biomarkers to proteotype *Campylobacter jejuni*. Using 10 strains belonging to *Campylobacter* spp. available from culture collections and 41 *Campylobacter jejuni* strains isolated from humans and foods, the ribosomal protein subunits L36, L32, S14, L24, L23, L7/L12, and S11 could be selected as the effective biomarkers for the proteotyping of *C. jejuni* at MALDI-TOF MS. An accurate database of their theoretical mass-based values was constructed by matching these gene DNA sequences and the observed mass peaks. We attempted to automatically classify 41 strains isolated from nature using this database and Strain Solution ver. 2 software, and 38 strains (93%) were correctly classified into the intended group based on the theoretical mass-based values. Thus, the seven biomarkers found in this study and Strain Solution ver. 2 are promising for the proteotyping of *C. jejuni* by MALDI-TOF MS.

## 1. Introduction

*Campylobacter* spp. are microaerophilic, non-spore-forming, Gram-negative helical rods, of which 33 species have been reported [1]. *Campylobacter jejuni* and *Campylobacter coli* have been the most common causative agents of *Campylobacter* infections in humans for about 100 years. *Campylobacter jejuni*/*coli* infections, commonly associated with gastroenteritis and diarrhea, are still increasing in many industrialized countries [2]. In severe cases, *C. jejuni* infections are thought to cause rare complications including neuropathy, such as Guillain–Barré syndrome and Fisher syndrome. *C. jejuni*/*coli* is widely distributed in the intestinal tracts of livestock, poultry, pets, and wild animals, and bacteria thought to originate from them have been isolated from rivers, lakes, and even sewage [3]. Chickens are the most significant source of *C. jejuni*/*coli* infection in developed countries, and its contamination rate in chicken is much higher than that of other livestock [4]. To reduce such food poisoning caused by chicken, it is important to monitor and control *C. jejuni*/*coli* in poultry processing [5].

Therefore, there are a variety of diagnostics analysis tools for determining *Campylobacter* by genus and species, and further classification. These include biochemical analysis based on traditional culturing [6], genetic approaches such as multiplex-PCR [7,8], pulsed-field gel electrophoresis (PFGE) [9], multi-locus sequence typing (MLST) focusing on seven housekeeping genes [10,11], and immunochemical approaches. For the past 30 years, two serotyping methods (Penner and Lior types) have been used in epidemiological studies of *C. jejuni* and *C. coli* [12,13]. *Campylobacter* spp. lack lipopolysaccharide in the outer-cell membrane and instead express lipooligosaccharide and capsule polysaccharide. The primary serotyping by Penner is thought to reflect the differentiation of the capsule polysaccharide [14,15]. Penner and Lior serotyping is useful for tracing the source of contamination, though agglutination tests using antisera involve complicated and time-consuming operations, and require skilled manipulation. More recently, whole-genome sequencing (WGS) technologies have been applied clinically as a robust alternative to conventional typing methods such as PFGE and MLST [16,17]. Using the genetic information available from WGS analysis, Cody et al. found the core genome of 1343 loci in *C. jejuni* and proposed the core genome MLST (cgMLST), which enables the high-resolution analysis of *C. jejuni*.

However, in terms of human health protection and food safety, a crucial key for the diagnosis and prevention of foodborne illness has been the establishment of a rapid and accurate discrimination method of foodborne pathogens at the strain or serovar level. In recent years, microbial classification using matrix-assisted laser desorption/ionization time-of-flight mass spectrometry (MALDI-TOF MS) has been rapidly gaining popularity in the clinical and food industries [18]. With this method, it is easy to obtain analytical results and to analyze multiple samples in a short time, thus enabling simple and rapid identification and typing of microorganisms. To overcome the limit of the conventional serotyping of *Campylobacter* spp., several research groups have developed a MALDI-TOF MS-based identification of *Campylobacter* spp. [19,20,21,22]. Mandrell et al. reported that six *Campylobacter* species (*C. coli*, *C. jejuni*, *C. lali*, *C. specterum*, *C. helveticus*, and *C. upsaliensi*) could be distinguished using biomarkers, ribosomal protein L7/L12, DNA-binding protein HU/HCj, ribosomal protein S13, chaperonin GroES, unknown protein of 9651 Da, 12,786 Da, and 9796 Da. In addition, Zautner et al. reported grouping results of *C.jejuni* by MALDI-TOF MS-reflected phenotypic aspects clustered by MLST [23]. More recently, MALDI-TOF MS combined with machine learning showed that the sensitive and specific prediction of *C. jejuni* Sequence type (ST) was possible [24].

The *S10-spc-alpha* operon gene-encoded ribosomal protein mass spectrum (*S10*-GERMS) method is one of the MALDI-TOF MS microbial identification and discrimination approaches, which provides a theoretical mass-based database for serotype- or species-specific biomarkers [25,26,27]. By combining this method and a proteotyping software Strain Solution ver. 2, which is created from the collaboration with Meijo University and Shimadzu corporation, we demonstrated the advanced classification of food-borne-related bacteria such as *Escherichia coli*, *Listeria monocytogenes*, and *Salmonella enterica* [27,28,29].

In this study, we constructed a theoretical mass-based database allowing the proteotyping of *Campylobacter* spp. and demonstrated proteotyping of 41 isolates of *C. jejuni* using the Strain Solution ver. 2 software.

## 2. Materials and Methods

### 2.1. Bacterial Strains

The *Campylobacter* spp. used in this study are summarized in Table 1. The strains obtained from public culture collections, American Type Culture Collection (ATCC) and Japan Collection of Microorganisms (JCM), were used to construct the theoretical MS database. Forty-one strains from No. 11 to 51 in Table 1 isolated from food and humans and preserved at the Osaka Institute of Public Health in Japan were used for the evaluation of the constructed database. ST of MLST was determined by PCR and Sanger sequencing with DNA primers listed in Table S1, which were designed according to the open access PubMLST website (http://pubmlst.org/campylobacter/info/primers.shtml (accessed on 14th December 2016) [30]. The Penner serotype of *C. jejuni* subsp. *jejuni* was determined by antisera, commercially available from Denka (Tokyo, Japan) [31]. Penner genetic typing was conducted according to the modified Capsule PCR multiplex typing system developed by Konno et al. [32], of which the original scheme was described by Poly et al. [33].

### 2.2. Construction of the Mass Database

Theoretical mass values of ribosomal proteins encoded in the *S10-spc-alpha* operon were calculated based on genetic information obtained by DNA sequencing with the primers listed in Table S2. DNA sequences determined in this study and selected as working biomarkers in MALDI-TOF MS were deposited to the DNA Data Bank of Japan (DDBJ) and are available in the DDBJ/EMBL/GenBank databases under accession numbers LC723726-LC723765.

### 2.3. MALDI-TOF MS Analysis

All strains were cultured on a 14EG medium plate (https://www.jcm.riken.jp/cgi-bin/jcm/jcm_grmd?GRMD=14 (accessed on 10th December 2016) recommended by JCM under a microaerobic condition (6–12% O_2_, 5–8% CO_2_) at 37 °C for two nights using Aneropack (Sugiyamagen, Tokyo, Japan). The bacterial colonies grown on the agar plates were picked by disposable loops and mixed with 10 μL of matrix solution (25 mg/mL sinapinic acid [Fujifilm Wako Pure Chemicals, Osaka, Japan], 0.6 (*v*/*v*)% trifluoroacetic acid, and 50 (*v*/*v*)% acetonitrile). The mixtures (1.2 μL) were spotted on the wells of a dry metal analytical plate which were precoated with 0.5 μL of saturated sinapinic acid dissolved in ethanol. After drying in air, they were analyzed with a positive linear mode by the AXIMA MALDI-TOF MS system for microorganisms (Shimadzu corporation, Kyoto, Japan) and the SARAMIS database v 3.5 (VITEK MS, bioMérieux, Marcy l’Etoile, France), which utilizes a fingerprinting approach. To further precisely analyze in Strain Solution 2, MS data categorized as *C. jejuni* in the SARAMIS database were calibrated with the common *m*/*z* peaks distributed in *C. jejuni*, namely *m*/*z* 4365.42 (ribosomal protein L36), 6826.31 (S14), 7034.49 (L29), 10,323.07 (S19), 11,673.73 (S10), and 16,376.32 (L16). Then, the analytical data of mass values and intensities were scanned on Strain Solution ver. 2 software at the tolerance of 800 ppm and with auto peak selection mode.

## 3. Results

### 3.1. Construction of the Theoretical MS Database

We obtained 10 strains of *Campylobacter* spp. and found that all of the ribosomal proteins encoded in *S10-spc-alpha* operon had sequence diversity according to the species or serotypes (Table 2, please refer to the registered DNA sequences described in Section 2.2). Among these, we could observe mass peaks derived from 15 kinds of ribosomal proteins (subunit S10, L23, L22, L16, L29, S17, L14, L24, S14, L18, L15, L36, and S11 in *S10-spc-alpha* operon) and additional biomarker candidates L32 and L7/L12, however, S10, L22, L16, L29, S17, L14, L18, L15, and S13 were not suitable as biomarkers because unknown protein mass peaks were overlapped on these mass peaks (data not shown).

Therefore, based on the previous results, we selected seven biomarkers as shown in Table 3. The ribosomal S11 peaks, except for *C. fetus* subsp. *Fetus*, were observed at the methylated (+14) mass values. The S11 peaks of *C. jejuni* ATCC700819 were not found, whereas it had a corresponding gene whose methylated theoretical mass value was same as that of other *C. jejuni* subsp. *jejuni*. In addition, the isolates from foods or humans were analyzed by MALDI-TOF MS, and we found some peaks showing characteristic mass values different from those of the strains obtained from the culture collection. Therefore, the gene sequences corresponding to the peaks were analyzed and included in the database. Sequence analysis revealed that some strains isolated from foods or humans had unique theoretical values that were not present in the culture collection strains. Therefore, five strains (C15_93, C15_94, C15_97, C15_113, C14_188) were selected as representatives of each group to be added to the database. Finally, the theoretical mass-based value database including these five strains was constructed.

### 3.2. Strain Solution Ver. 2 Analysis

**Figure 1:**
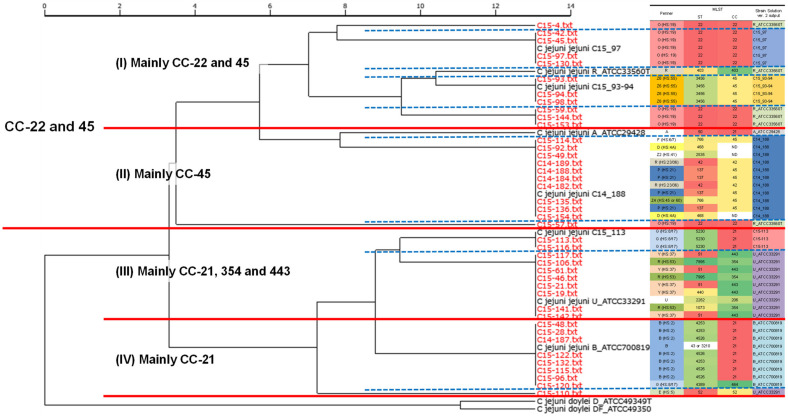
Cluster analysis with the result of Strain Solution ver. 2. The mass data were scanned with Strain Solution ver. 2 with the registered protein markers shown in Table 3. The clustering tree was drawn with UPGMA method using the same software. The red lines and blue dotted lines indicate that there are four major groups and further subdivisions, respectively. Each group is shown in a different color. ND: not determined.

First, all *Campylobacter* spp. strains used in this study were identified at the species level by the SARAMIS fingerprinting method. Based on the constructed and registered database with seven biomarkers on the Strain Solution ver. 2 software (Table 3), the MALDI-TOF MS analysis data (*m*/*z* and intensity) of each strain were scanned. The biomarker hit scores and output results are summarized in Table 4. Among 41 strains isolated from food or humans, 6 or 7 biomarkers in 38 strains were detected and correctly proteotyped as the intended groups according to the theoretical mass values. Though three strains, namely C15-92, C15-135, and C15-141, showed the hit score 7 (100% matching) to the correct groups, they matched another group with the same score. Therefore, the correct classification rate was 93% (38/41). To further evaluate the results, a phylogenetic tree was drawn based on all detected biomarker peaks in the Strain Solution ver. 2 software (Figure 1). By comparing Penner serotype, ST, and clonal complex (CC) with this proteotyping phylogenetic tree, it was found that most of the strains with CC type 22, 21, and 45 separated each other and those with the same CC type were assigned into the clusters. For Penner serotypes (Penner genetic typing), G (HS: 8/17) and B (HS: 2) were clearly distinguished from the others.

## 4. Discussion

In this study, we constructed a theoretical mass-based database for the proteotyping of *Campylobacter* species by MALDI-TOF MS and Strain Solution ver. 2 software. The selected biomarker peaks were all derived from ribosomal protein subunits, and our previously reported proteotyping approach allowed us to construct a widely disseminated theoretical *m*/*z* value database (Table 3). Although useful biomarkers for the identification of *Campylobacter* using MALDI-TOF MS have been vigorously investigated by Mandrell et al. [19], it should be noted that the biomarkers other than ribosomal protein L7/L12 are first reported in this study. The reasons we could find new biomarkers in this study would be that we selected stably detected biomarker peaks either with small intensity or molecular weights greater than *m*/*z* 13,000. The 41 strains isolated from the foods and humans were used to evaluate the detection of the selected seven biomarker peaks and the validity of this database, and generally we could get more than six marker hits in reference to the database, allowing classification into the intended groups based on the theoretical mass-based value database (Table 4). As for the three strains doubly hitting the correct and the other groups with the same score of 7, adding a new biomarker to the database could help with accurate identification. However, at present, since we have not found any biomarkers other than Table 3 that can be detected stably, the sample preparation method may need to be improved, as examined for *Salmonella enterica* [34].

Several attempts to correlate STs by MLST with MALDI-TOF MS results have been reported, but the large number and complexity of the MLST makes it difficult to correlate all types [24,35]. In this study, we determined Penner serotypes including genetic types, STs, and CC, and compared them with MALDI-TOF MS proteotyping results. The cluster analysis of the MALDI-TOF MS results using the obtained phylogenetic tree on Strain Solution ver. 2 showed that the tested *C. jejuni* strains divided into two main clusters. One consisted of mainly CC-22 and CC-45, and the other was CC-21 and the others such as CC-354 and 443, and these main clusters further classified into two sub-clusters (Figure 1). In total, we could observe four distinct clusters shown as I to IV in Figure 1. Furthermore, group I was subdivided into five, and group II into three clusters. Focusing on the Penner serotypes, we can see that O (HS: 19) and Z_6_ (HS: 55) types tend to belong to cluster I, P (HS: 21), D (HS: 4A), R (HS: 23/36), and others to cluster II, G (HS: 8/17), Y (HS: 37), and R (HS: 53) to cluster III. The Penner type of cluster IV, to which CC-21 mainly belongs, was found to be mainly B (HS: 2). The major *C. jejuni* strains isolated from humans are CC-21 and 45, but CC-21 is more resistant to environmental stresses such as high temperatures and freezing, and has acquired resistance to various drugs more frequently [36,37]. Therefore, the clear separation of CC-21 and 45 in the present results indicates that such differences may be reflected in the peak appearance of MALDI-TOF MS, which was also supposed by a previous report [35]. This suggests that Strain Solution ver. 2 analysis may have the possibility of classification by lineage like *Listeria monocytogenes* described previously [28].

In addition, we have found CC-specific biomarker candidate peaks by MALDI-TOF MS analysis as follows: *m*/*z* 2868 (CC-354), *m*/*z* 3216 (CC-468), *m*/*z* 4158 (CC-21), *m*/*z* 4866 (CC-354), *m*/*z* 5244 (CC-354), *m*/*z* 5267 (CC-21), *m*/*z* 6136 (CC-468), *m*/*z* 8256 (CC-354), *m*/*z* 9256 (CC-443), *m*/*z* 9195 (CC-42), *m*/*z* 9209 (CC-45), *m*/*z* 9803 (CC-21), *m*/*z* 11,219 (CC-21), *m*/*z* 12,012 (CC-42), *m*/*z* 13,247 (CC-21 and 464), *m*/*z* 14,088 (CC-468), *m*/*z* 14,615 (CC-45), *m*/*z* 14,698 (CC-21), *m*/*z* 15,635 (CC-45), *m*/*z* 16,225 (CC-21), *m*/*z* 18,080 (CC-468), *m*/*z* 18,491 (CC-354), *m*/*z* 18,533 (CC-21), and *m*/*z* 20,740 (CC-21) (data not shown). With further increased validation and improvement by accumulating analyses of biomarkers, more detailed classification may be possible. The theoretical mass-based database which is useful for the proteotyping of *C. jejuni* as well as the identification of *Campylobacter* spp. by MALDI-TOF MS will be broadly used in clinical and food-related investigations, allowing a rapid MALDI analysis to make a quicker decision before the CC or Penner serotypes, which can contribute to *Campylobacter* risk management.

WGS has rapidly developed because of the prevailing technology of the next-generation sequencing. The enrichment of the web-accessible WGS database will also make possible the creation of a sophisticated method for the establishment of a database constructed with genetically theoretical mass-based biomarkers, leading to the proteotyping of bacterial isolates at the species, strain, and serovar level. The conceptual principle using genetically theoretical mass-based biomarkers is valid for the MALDI-TOF MS analysis with machine learning, and will pave the way for greater change.

## 5. Patents

Patent WO2017168741 is resulting from the work reported in this manuscript.

## Figures and Tables

**Table 1:** *Campylobacter* spp. strains used in this study.

No.	Strain No.	Source	Species	MLST Sequence Type (Clonal Complex)	Penner Serotype (Genetic Type) *
1	ATCC33560^T^		*jejuni*	403 (CC-403)	R
2	ATCC29428		*jejuni*	50 (CC-21)	A
3	ATCC33291		*jejuni*	2282 (CC-206)	U
4	ATCC700819		*jejuni*	ND **	B
5	ATCC49349^T^		*jejuni*	62	
6	ATCC49350		*jejuni*	1840	
7	JCM2529		*coli*	900	
8	JCM2527		*fetus*		
9	JCM2528		*fetus*		
10	JCM14870		*lari*		
11	C15-92	Food	*jejuni*	468 (ND)	D (HS: 4A)
12	C15-154	Human	*jejuni*	468 (ND)	D (HS: 4A)
13	C15-141	Food	*jejuni*	1073 (CC-354)	R (HS: 53)
14	C14-184	Human	*jejuni*	137 (CC-45)	P (HS: 21)
15	C14-188	Human	*jejuni*	137 (CC-45)	P (HS: 21)
16	C15-136	Human	*jejuni*	137 (CC-45)	P (HS: 21)
17	C15-4	Human	*jejuni*	22 (CC-22)	O (HS: 19)
18	C15-42	Human	*jejuni*	22 (CC-22)	O (HS: 19)
19	C15-45	Human	*jejuni*	22 (CC-22)	O (HS: 19)
20	C15-57	Human	*jejuni*	22 (CC-22)	O (HS: 19)
21	C15-59	Human	*jejuni*	22 (CC-22)	O (HS: 19)
22	C15-97	Human	*jejuni*	22 (CC-22)	O (HS: 19)
23	C15-130	Human	*jejuni*	22 (CC-22)	O (HS: 19)
24	C15-144	Human	*jejuni*	22 (CC-22)	O (HS: 19)
25	C15-153	Human	*jejuni*	22 (CC-22)	O (HS: 19)
26	C15-49	Human	*jejuni*	2535 (ND)	Z_2_ (HS: 41)
27	C15-93	Food	*jejuni*	3456 (CC-45)	Z_6_ (HS: 55)
28	C15-94	Food	*jejuni*	3456 (CC-45)	Z_6_ (HS: 55)
29	C15-98	Human	*jejuni*	3456 (CC-45)	Z_6_ (HS: 55)
30	C14-182	Human	*jejuni*	42 (CC-42)	R (HS: 23/36)
31	C14-189	Human	*jejuni*	42 (CC-42)	R (HS: 23/36)
32	C15-28	Human	*jejuni*	4253 (CC-21)	B (HS: 2)
33	C15-48	Human	*jejuni*	4253 (CC-21)	B (HS: 2)
34	C15-132	Human	*jejuni*	4253 (CC-21)	B (HS: 2)
35	C15-120	Food	*jejuni*	4389 (CC-464)	G (HS: 8/17)
36	C15-19	Human	*jejuni*	440 (CC-443)	Y (HS: 37)
37	C14-187	Human	*jejuni*	4526 (CC-21)	B (HS: 2)
38	C15-96	Food	*jejuni*	4526 (CC-21)	B (HS: 2)
39	C15-122	Human	*jejuni*	4526 (CC-21)	B (HS: 2)
40	C15-115	Food	*jejuni*	4526 (CC-21)	B (HS: 2)
41	C15-21	Human	*jejuni*	51 (CC-443)	Y (HS: 37)
42	C15-61	Human	*jejuni*	51 (CC-443)	Y (HS: 37)
43	C15-117	Food	*jejuni*	51 (CC-443)	Y (HS: 37)
44	C15-142	Food	*jejuni*	51 (CC-443)	Y (HS: 37)
45	C15-110	Human	*jejuni*	52 (CC-52)	E (HS: 5)
46	C15-113	Food	*jejuni*	5230 (CC-21)	G (HS: 8/17)
47	C15-116	Food	*jejuni*	5230 (CC-21)	G (HS: 8/17)
48	C15-114	Food	*jejuni*	766 (CC-45)	F (HS: 6/7)
49	C15-135	Human	*jejuni*	766 (CC-45)	Z_4_ (HS: 45 or 60)
50	C15-46	Human	*jejuni*	7995 (CC-354)	R (HS: 53)
51	C15-106	Human	*jejuni*	7995 (CC-354)	R (HS: 53)

A superscript T indicates that the strain is the type strain. ND; not determined. * Penner genetic typing determined by modified Capsule PCR multiplex typing system. ** The ST of the strain ATCC700819 may be a new type because it did not exactly match the database-registered type, but 6 in 7 genes matched against ST 43 or 3210 types.

**Table 2:** Theoretical masses of biomarker peaks in *S10-spc-alpha* operon and their detection.

	Strain	ATCC 33560^T^	ATCC 29428	ATCC 33291	ATCC 700819	ATCC 49349^T^	ATCC 49350	JCM 2529	JCM 2527	JCM 2528	JCM 14870
	spp.	*jejuni*	*jejuni*	*jejuni*	*jejuni*	*jejuni*	*jejuni*	*coli*	*fetus*	*fetus*	*lari*
	subsp.	*jejuni*	*jejuni*	*jejuni*	*jejuni*	*doylei*	*doylei*		*fetus*	*venerealis*	*lari*
		Penner serotype									
Marker protein	Detection of the peak	R	A	U	B						
S10	+	11,673.73	11,673.73	11,673.73	11,673.73	11,673.73	11,673.73	11,737.77	11,676.71	11,676.71	11,704.76
L3	+/−	20,723.83	20,723.83	20,737.85	20,737.85	20,723.83	20,735.88	20,723.83	20,470.44	20,470.44	20,648.78
L4	−	22,025.25	22,071.34	22,071.34	22,125.43	22,105.36	22,071.34	22,061.30	22,011.06	22,012.04	22,253.54
L23	+	10,424.11	10,465.20	10,437.15	10,437.15	10,394.08	10,394.08	10,394.08	10,375.08	10,375.08	10,480.13
L2	−	30,417.29	30,290.10	30,290.10	30,276.03	30,304.13	30,304.13	30,290.10	30,137.88	30,121.88	30,290.04
S19	+	10,323.07	10,323.07	10,323.07	10,323.07	10,323.07	10,334.19	10,335.13	10,278.10	10,278.10	10,306.13
L22	+	15,621.46	15,112.83	15,112.83	15,112.83	15,124.89	15,124.89	14,436.07	11,762.69	11,762.69	13,194.47
S3	−	25,933.20	25,933.20	25,933.20	25,933.20	25,923.17	25,939.16	25,910.14	25,790.02	25,790.02	25,875.16
L16	+	16,376.32	16,376.32	16,376.32	16,376.32	16,376.32	16,376.32	16,376.32	16,213.05	16,213.05	16,388.33
L29	+	7034.49	7034.49	7034.49	7034.49	7034.49	7034.49	7006.48	6894.22	6894.22	6994.42
S17	+	9419.19	9419.19	9419.19	9419.19	9419.23	9419.23	9405.17	9607.34	9607.34	9446.26
L14	+	13,306.74	13,306.74	13,306.74	13,306.74	13,306.74	13,306.74	13,306.74	13,314.82	13,314.82	13,277.74
L24	+	8152.75	8152.75	8152.75	8152.75	8179.78	8179.78	8152.75	8027.58	8027.58	8042.64
L5	+/−	20,214.81	20,214.81	20,214.81	20,214.81	20,214.81	20,228.84	20,199.80	19,979.42	19,979.42	20,264.92
S14	+	6826.31	6826.31	6826.31	6826.31	6826.31	6826.31	6812.28	6729.15	6729.15	6785.22
S8	+/−	14,734.14	14,734.14	14,734.14	14,734.14	14,690.13	14,691.07	14,753.18	14,755.94	14,755.94	14,738.13
L6	+/−	19,456.44	19,456.44	19,456.44	19,456.44	19,455.46	19,407.42	19,529.49	19,259.12	19,259.12	19,422.47
L18	+	13,239.71	13,239.71	13,267.76	13,267.76	13,239.71	13,267.76	13,267.72	12,710.93	12,710.93	13,100.57
S5	+/−	15,797.38	15,797.38	15,797.38	15,797.38	15,604.11	15,797.38	15,797.38	15,768.45	15,768.45	15,823.42
L15	+	14,028.27	14,028.27	14,028.27	14,028.27	14,028.27	14,028.27	13,923.18	14,229.21	14,229.21	14,221.43
L36	+	4365.42	4365.42	4365.42	4365.42	4365.42	4365.42	4365.42	4332.38	4332.38	4365.42
S13	+	13,604.90	13,604.90	13,604.90	13,604.90	13,604.90	13,604.90	13,606.87	13,669.93	13,669.93	13,596.84
S11	+	13,777.11 (Me)	13,777.11 (Me)	13,777.11 (Me)	ND	13,807.14 (Me)	13,807.14 (Me)	13,777.11 (Me)	13,819.18	13,833.18 (Me)	13,763.08 (Me)
S4	−	23,779.54	23,779.54	23,765.52	23,779.54	23,765.52	23,765.52	23,765.52	23,742.42	23,742.42	23,839.57
L32	+	5483.43	5497.46	5497.46	5497.46	5483.43	5513.50	5510.46	5531.48	5531.48	5537.57
L7/L12	+	12,886.70	12,886.70	12,900.73	12,942.81	12,886.70	12,886.70	12,854.71	12,869.71	12,869.71	12,968.81

+: detected. +/−: peak intensity is weak. −: not detected. ND: no corresponding gene found. (Me) indicates methylated value.

**Table 3:** Selected biomarkers for proteotyping of *Campylobacter* spp. in MALDI-TOF MS.

				Biomarker						
Species	Subspecies	Penner Serotype	Strain	L36	L32	S14	L24	L23	L7/L12	S11 ^a^
*jejuni*	*jejuni*	R	ATCC33560^T^	4365.42 (I)	5483.43 (I)	6826.31 (I)	8152.75 (I)	10,424.11 (I)	12,886.7 (I)	13,777.11 (I)
	*jejuni*	A	ATCC29428	4365.42 (I)	5497.46 (II)	6826.31 (I)	8152.75 (I)	10,465.2 (III)	12,886.7 (I)	13,777.11 (I)
	*jejuni*	U	ATCC33291	4365.42 (I)	5497.46 (II)	6826.31 (I)	8152.75 (I)	10,437.15 (II)	12900.73 (II)	13,777.11 (I)
	*jejuni*	B	ATCC700819	4365.42 (I)	5497.46 (II)	6826.31 (I)	8152.75 (I)	10,437.15 (II)	12,942.81 (V)	13,777.11 ^b^ (I)
	*doylei*		ATCC49349^T^	4365.42 (I)	5483.43 (I)	6826.31 (I)	8179.78 (II)	10,394.08 (IV)	12,886.7 (I)	13,807.14 (IV)
	*doylei*		ATCC49350	4365.42 (I)	5513.5 (III)	6826.31 (I)	8179.78 (II)	10,394.08 (IV)	12,886.7 (I)	13,807.14 (IV)
	*jejuni*	Z_6_	C15_93, 94	4365.42 (I)	5524.49 (VII)	6826.31 (I)	8152.75 (I)	10424.11 (I)	12,886.7 (I)	13,777.11 (I)
	*jejuni*	O	C15_97	4365.42 (I)	5497.46 (II)	6826.31 (I)	8152.75 (I)	10,424.11 (I)	12,916.73 (VII)	13,777.11 (I)
	*jejuni*	G	C15_113	4365.42 (I)	5497.46 (II)	6826.31 (I)	8152.75 (I)	10,437.15 (II)	13,173.14 (VIII)	13,777.11 (I)
	*jejuni*	P	C14_188	4365.42 (I)	5497.46 (II)	6826.31 (I)	8152.75 (I)	10,424.11 (I)	12,886.7 (I)	13,777.11 (I)
*coli*	-		JCM2529	4365.42 (I)	5510.46 (IV)	6812.28 (II)	8152.75 (I)	10,394.08 (IV)	12,854.71 (III)	13,777.11 (I)
*fetus*	*fetus*		JCM2527	4332.38 (II)	5531.48 (V)	6729.15 (III)	8027.58 (III)	10,375.08 (V)	12,869.71 (IV)	13,819.18 (III)
	*venerealis*		JCM2528	4332.38 (II)	5531.48 (V)	6729.15 (III)	8027.58 (III)	10,375.08 (V)	12,869.71 (IV)	13,833.18 (V)
*lari*	*lari*		JCM14870	4365.42 (I)	5537.57 (VI)	6785.22 (IV)	8042.64 (IV)	10,480.13 (VI)	12,968.81 (VI)	13,763.11 (II)

^a^, Methylated values observed in the mass spectra are shown, except *C. fetus* sbsp. *fetus.* ^b^, the corresponding mass peak was not found. Different mass patterns of each biomarker are indicated as Roman numerals in the parentheses.

**Table 4:** Strain Solution ver. 2 analysis result.

Analyte	Hit Score	Phenotypic Cluster	Output Strain Name	Summary
C15-4	7	*jejuni*	R_ATCC33560^T^	correct
C15-19	7	*jejuni*	U_ATCC33291	correct
C15-21	7	*jejuni*	U_ATCC33291	correct
C15-28	7	*jejuni*	B_ATCC700819	correct
C15-42	7	*jejuni*	C15_97	correct
C15-45	7	*jejuni*	C15_97	correct
C15-46	7	*jejuni*	U_ATCC33291	correct
C15-48	7	*jejuni*	B_ATCC700819	correct
C15-49	7	*jejuni*	C14_188	correct
C15-57	6	*jejuni*	C15_97	correct
C15-59	7	*jejuni*	R_ATCC33560^T^	correct
C15-61	7	*jejuni*	U_ATCC33291	correct
C15-92	7	*jejuni*	C14_188 and C15_93094	double hit
C15-93	7	*jejuni*	C15_93-94	correct
C15-94	7	*jejuni*	C15_93-94	correct
C15-96	7	*jejuni*	B_ATCC700819	correct
C15-97	7	*jejuni*	C15_97	correct
C15-98	7	*jejuni*	C15_93-94	correct
C15-106	7	*jejuni*	U_ATCC33291	correct
C15-110	6	*jejuni*	U_ATCC33291	correct
C15-113	7	*jejuni*	C15_113	correct
C15-114	7	*jejuni*	C14_188	correct
C15-115	7	*jejuni*	B_ATCC700819	correct
C15-116	7	*jejuni*	C15_113	correct
C15-117	7	*jejuni*	U_ATCC33291	correct
C15-120	7	*jejuni*	B_ATCC700819	correct
C15-122	7	*jejuni*	B_ATCC700819	correct
C15-130	7	*jejuni*	C15_97	correct
C15-132	7	*jejuni*	B_ATCC700819	correct
C15-135	7	*jejuni*	C14_188 and C15_93-94	double hit
C15-136	7	*jejuni*	C14_188	correct
C15-141	7	*jejuni*	B_ATCC700819 and U_ATCC33291	double hit
C15-142	7	*jejuni*	U_ATCC33291	correct
C15-144	7	*jejuni*	R_ATCC33560^T^	correct
C15-153	7	*jejuni*	R_ATCC33560^T^	correct
C15-154	7	*jejuni*	C14_188	correct
C14-182	7	*jejuni*	C14_188	correct
C14-184	7	*jejuni*	C14_188	correct
C14-187	7	*jejuni*	B_ATCC700819	correct
C14-188	7	*jejuni*	C14_188	correct
C14-189	7	*jejuni*	C14_188	correct

Double hit means two output results were obtained with identical scores.

## Data Availability

The datasets presented in this study can be found in this article/Supplementary Materials, and DDBJ/EMBL/GenBank. Further information and requests for resources and reagents should be directed to and will be fulfilled by the corresponding authors.

## Supplementary Materials

The following supporting information can be downloaded at: https://www.mdpi.com/article/10.3390/microorganisms11010202/s1, Table S1: DNA primers used for MLST; Table S2: DNA primers used for sequencing of biomarkers.
