# Supplementary material for: Proteotyping of Campylobacter jejuni by MALDI-TOF MS and Strain Solution Version 2 Software
Source: Microorganisms. 2023 Jan 12;11(1):202. doi: 10.3390/microorganisms11010202 (PMC9866418; doi:10.3390/microorganisms11010202)
Supplement: Supplementary file 1 [file microorganisms-11-00202-s001.zip › microorganisms-2128625-supplementary.pdf]

Supplementary Material

Supplementary Table S1. DNA primers used for MLST

Primer name	Sequence (5'→3')	Target	Gene	Purpose
Cj_aspA_A9-F	AGTACTAATGATGCTTATCC	*C. jejuni*	*aspA* (aspatase)	amplification
Cj_aspA_A10-R	ATTTCATCAATTTGTTCTTTGC			
Cj_glnA_A1-F	TAGGAACTTGGCATCATATTACC		*glnA* (glutamine synthetase)	
Cj_glnA_A2-R	TTGGACGAGCTTCTACTGGC			
Cj_gltA_A1-F	GGGCTTGACTTCTACAGCTACTTG		*gltA* (citrate synthase)	
Cj_gltA_A2-R	CCAAATAAAGTTGTCTTGGACGG			
Cj_glyA_A1-F	GAGTTAGAGCGTCAATGTGAAGG		*glyA* (serine hydroxy methyl transferase)	
Cj_glyA_A2-R	AAACCTCTGGCAGTAAGGGC			
Cj_pgm_A7-F	TACTAATAATATCTTAGTAGG		*pgm* (phospho glucomutase)	
Cj_pgm_A8-R	CACAACATTTTTCATTTCTTTTTC			
Cj_tkt_A3-F	GCAAACTCAGGACACCCAGG		*tkt* (transketolase)	
Cj_tkt_A6-R	AAAGCATTGTTAATGGCTGC			
Cj_uncA_A7-F	ATGGACTTAAGAATATTATGGC		*uncA* (ATP synthase alpha subunit)	
Cj_uncA_A8-R	ATAAATTCCATCTTCAAATTCC			
Cj_aspA_S3-F	CCAACTGCAAGATGCTGTACC		*aspA* (aspatase)	sequencing
Cj_aspA_S6-R	TTCATTTGCGGTAATACCATC			
Cj_glnA_S3-F	CATGCAATCAATGAAGAAAC		*glnA* (glutamine synthetase)	
Cj_glnA_S6-R	TTCCATAAGCTCATATGAAC			
Cj_gltA_S3-F	CTTATATTGATGGAGAAAATGG		*gltA* (citrate synthase)	
Cj_gltA_S6-R	CCAAAGCGCACCAATACCTG			
Cj_glyA_S5-F	GCTAATCAAGGTGTTTATAT		*glyA* (serine hydroxy methyl transferase)	
Cj_glyA_S7-F	AGCCTAATTCAGGTTCTCAA			
Cj_glyA_S4-R	AGGTGATTATCCGTTCCATCGC			
Cj_pgm_S5-F	GGTTTTAGATGTGGCTCATG		*pgm* (phospho glucomutase)	
Cj_pgm_S2-R	TCCAGAATAGCGAAATAAGG			
Cj_tkt_S5-F	GCTTAGCAGATATTTTAAGTG		*tkt* (transketolase)	
Cj_tkt_S6-R	AAGCCTGCTTGTTCTTTGGC			
Cj_uncA_S3-F	AAAGTACAGTGGCACAAGTGG		*uncA* (ATP synthase alpha subunit)	
Cj_uncA_S4-R	TGCCTCATCTAAATCACTAGC			
Aspcoli S1	CAACTTCAAGATGCAGTACC	*C. coli*	*aspA* (aspatase)	amplification & sequencing
Aspcoli S2	ATCTGCTAAAGTATGCATTGC			
Glncoli S1	TTCATGGATGGCAACCTATTG		*glnA* (glutamine synthetase)	
Glncoli S2	GCTTTGGCATAAAAGTTGCAG			
Gltcoli S1	GATGTAGTGCATCTTTTACTC		*gltA* (citrate synthase)	
Gltcoli S2	AAGCGCTCCAATACCTGCTG			

Glycoli S1	TCAAGGCGTTTATGCTGCAC	*glyA* (serine hydroxy methyl transferase)
Glycoli S2	CCATCACTTACAAGCTTATAC	
Pgmcoli S1	TTATAAGGTAGCTCCGACTG	*pgm* (phospho glucomutase)
Pgmcoli S2	GTTCCGAATAGCGAAATAACAC	
Tktcoli S1	AGGCTTGTGTTTTCAGGCGG	*tkt* (transketolase)
Tktcoli S2	TGACTTCCTTCAAGCTCTCC	
Unccoli S1	AAGCACAGTGGCTCAAGTTG	*uncA* (ATP synthase alpha subunit)
Unccoli S2	CTACTTGCCTCATCCAATCAC	

Supplementary Table S2. DNA primers used for sequencing of biomarkers

Primer Name		Sequence
**Cam-S10**	F	GGAAAGAATYAGGCTTAAGCTAAAAGCTTA
	R	TCCGGTGCWAGWGAWACRATYTTCATA
	1	GGAATAYATYGTAGAAAAAATHGGHATGAG
	2r	TACCTGGYTGAACRCGACCTG
	3	GTGGTGGTAAAAARCCWGGAGACAA
	4	GGACCAAADGCWACHGCRCC
	5	ATATACTCCAAGYAGAAGATWTATBACAGG
	6	CCVGTTTATRTHACWGAAAATCAYATMGG
	7r	TCTWGCYTTWGTTGGAGATARTCTTATGAA
	8	ATAGAAAYTGGGARTCWAGATGGTTTCC
	9	CTTATGGHAAYATAGGDRTWAAAGTDTGGAT
	10	AAAAGCTAAAAACWATGCARCTWACTAAYCC
	New-F	CTTGGTATGTTGAAGGCGGG
	New-R	CAAAGATACGAGTTCCGATAG
**Cam-spc**	F	ATGTGTATYAARGTTTTAGGBGGTAGYAAA
	R	AATTTGAGCYTCDATYTTTCTCATYGTRTC
	1	GARTTTGATATHAAAAAYCCTATGCTTATMCC
	2	CAATGATWGCAAAAGCDGCMCGCAA
	3	GCWTATTGGGGAACTTAYAGAGCTTTA
	4r	TTAGATGTYTTRCCRGCTTTGCGKAT
	5	AATTTGAAGAAGTAATCGTCGAYATCGG
	6r	ATTRACSCCMGGWACTGGMACATAA
**Cam-α**	F	AAAGTHGAACTHACRCCHTATAGYCTTGA
	R	AGCCRCGCATRCTATCRAATTCATG
	1	TAAGYGAAGATGARGCHGCDGCTAT
	2r	CCTTTTCTRTGTCTTAAGCCTCTAWAGC
	3	GAGGACCAGTWGARAAATTAGAAAGACG
**Cam-S15**	F	GATCAACAAAGCGTAGAATA
	R	CGCTAGCTTTGGTAAATAGC
**Cam_L7L12**	F	GTTGCAGATGTTGCTAAGG
	R	GTCAAACCTCTTTCCATGC
**Cam_L32**	F	CTTTTTGCAAGTGATGGTAT
	R	ATTTCCCACTAAGATAGC
